# Speciation determination of iron and its spatial and seasonal distribution in coastal river

**DOI:** 10.1038/s41598-018-20991-0

**Published:** 2018-02-07

**Authors:** Yun Zhu, Xueping Hu, Dawei Pan, Haitao Han, Mingyue Lin, Yan Lu, Chenchen Wang, Rilong Zhu

**Affiliations:** 1 Key Laboratory of Coastal Environmental Processes and Ecological Remediation, Yantai Institute of Coastal Zone Research, Chinese Academy of Sciences, Yantai, 264003 P.R. China; 2 University of Chinese Academy of Sciences, Beijing, 100049 P.R. China; 3 Hunan Environmental Monitoring Center Station, State Environmental Protection Key Laboratory of Monitoring for Heavy Metal Pollutants, Changsha, 410019 P.R. China

## Abstract

In this study, the speciation of iron (Fe), including total Fe (TFe) and acidified dissolved Fe (ADFe), was assessed by fast cathodic absorption stripping voltammetry, using a gold electrode and 2-(5-bromo-2-pyridylazo)-5-diethylaminophenol (5-Br-PADAP) as a novel complexing agent for Fe. The validity and accuracy of this method were compared with the standard spectrophotometry and tested by the standard samples. Under optimized conditions, the Fe response was linear within the range of 0.01 to 1 μM with a detection limit of 1.2 nM. To further validate this method, the variation in concentrations of TFe and ADFe were investigated at twelve sampling stations in a local coastal river, in both the dry and wet season. Additionally, to further understand the interaction between Fe and environmental factors, the relationships between the concentration of Fe species and dissolved oxygen (DO) and salinity were also discussed.

## Introduction

Iron (Fe) is an essential micronutrient for almost all organisms^1^. It is involved in metabolic processes, such as chlorophyll synthesis, nitrate reduction, detoxification of reactive oxygen species and electron transport in photosynthesis and respiration^2^. Fe is the fourth most abundant element in the Earth’s crust, after oxygen, silicon and aluminum^3^, with an abundance of 5~6%^4,5^. The Fe concentration in water is proportionally less due to its low solubility^6^, with reports of Fe concentrations in natural water sources ranging from 10^−6^ M in river water, 10^−6^–10^−9^ M in coastal water and 10^−11^ M in ocean water^7^. Total Fe pools include species such as Fe(II) (ferrous) and Fe(III) (ferric) Fe, organically and inorganically complexed Fe, colloidal Fe and particulate Fe^8^. The concentration of Fe in the open oceans influences the content of carbon dioxide in the atmosphere and hence global climate^9^, with Fe concentrations in the ocean supplied from surrounding land and sediments. Fe is also an important biological and geochemical trace element in coastal environments and can affect marine organisms and ecosystems through atmospheric transmission and river transportation^10^.

Environmental deficiency of Fe can limit phytoplankton growth, as does limited supply of other nutrients such as phosphates, silicates and nitrates in some ocean areas^11^. In addition, the high concentration of Fe in surface waters can cause deterioration in the organoleptic properties of water, as well as interfering with water-treatment properties^12^. Excess Fe is of significant environmental concern due to its biogeochemical recycling and ecological risks^13^. Some studies have shown that the abundance of Fe may be a key contributory factor in the formation of red tides, along with nutrients such as nitrogen and phosphorus^14^. By understanding the source and role of total Fe, reduction in Fe supply can be used to reduce the probability of red tide events^15^. Therefore, the dynamic change of Fe in rivers, especially coastal rivers which flow into the ocean, have gained more attention in recent years, with the accurate determination of Fe in coastal rivers being of significant interest in environmental monitoring.

Many analytical methods had been developed to determine Fe species concentrations such as spectrophotometry^16,17^, atomic absorption spectrometry (AAS)^18,19^, inductively coupled plasma-mass spectrometry (ICP-MS)^20,21^ chemiluminescence^22–24^ and fluorescence^25^, among others. However, most of these methods require complex and expensive analytical equipment, limiting their potential application. Electrochemical methods have been identified as an extremely sensitive method for Fe determination, with various benefits, including simple equipment requirements, low costs, low detection limit, fast analysis speed^26–28^ and easily automated detection^29–31^. Several complexing agents have been used to accumulate Fe complexes onto electrode surfaces, such as 1-(2-piridylazo)-2-naphthol (PAN)^32^, 1-nitroso-2-naphthol (NN)^33^ and 2, 3-dihydroxynaphthalene (DHN)^34^. However, the chelating reaction times reported for these agents are relatively long, with many service restrictions.

**Figure 1:**
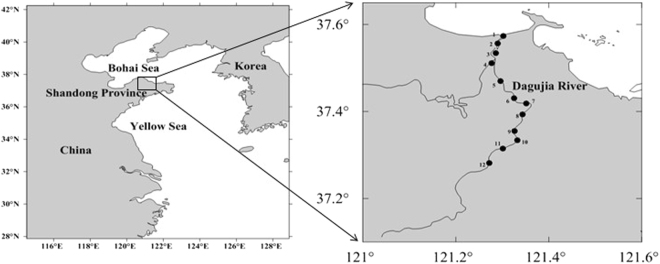
Sampling stations in the Dagujia River (Yantai, China). Maps were created with Surfer software, version 10.3.705, http://www.goldensoftware.com/products/surfer.

In this study, a gold electrode was used as a working electrode^35^ and 2-(5-bromo-2-pyridylazo)-5-diethylaminophenol (5-Br-PADAP) was used as a Fe complexing agent with a short chelating reaction time (<3 min)^36–38^. To further validate this method and investigate Fe biogeochemistry in coastal waters, the concentration and distribution of total Fe (TFe, acidified, <0.45 μm filtered, UV-digested for 30 minutes) and acidified dissolved Fe (ADFe, acidified, <0.45 μm filtered) in local coastal Dagujia River (Fig. 1), were investigated. Additionally, the relationships between the concentration of Fe with both DO and salinity were studied. The relationships between the concentrations of ADFe, TFe and DO were found to be negatively correlated, while no obvious relationship was observed between the concentrations of ADFe, TFe and salinity, in the studied area. Data shows that this method is reliable, providing a straight-forward and highly applicable method for the technical determination of the distribution of Fe in coastal waters.

## Method

### Reagents and solutions

Fe standard solutions were prepared from Fe chloride (Sinopharm Chemical Reagent Co., Ltd., China.) in 0.1 M HCl. HCl was of guaranteed reagent grade. 5-Br-PADAP was purchased from Aladdin Industrial Corporation. All other chemicals were of analytical reagent grade. Deionized water (18.2 MΩ cm specific resistance) was used throughout, obtained using a Pall Cascada laboratory water system. The Fe standard sample applied was GSB 07-1188-2000 standard.

### Apparatus

Salinity, DO, temperature and electrical conductivity of environmental water samples were measured using a multi-parameter controller (YSI 556MPS). All electrochemical experiments were carried out using a conventional three-electrode cell controlled by Electrochemical Work Station (CHI 660D, CH Instruments, Inc.). A gold disk electrode (3 mm in diameter) was used as the working electrode, with Ag/AgCl and platinum foil serving as the reference and counter electrodes, respectively. All potential values provided refer to Ag/AgCl. pH measurements were performed using a Model pH meter (E-201-C, Shanghai Leici Instrument Factory, China). UV-digestion was performed using a UV digester (Metrohm MVA-UV 705, Switzerland). A spectrophotometer (T6, Beijing Purkinje General Instrument Co., Ltd) was used for comparative Fe species concentration testing.

### Detection procedure

Unless otherwise stated, the experiments were performed in 0.1 M acetate buffer (pH 6.0) as the supporting electrolyte, containing 20 μM 5-Br-PADAP. Fe(III)-5-Br-PADAP complex was accumulated onto the surface of the Au electrode under a potential of −0.1 V for 360 s, with continual stirring. Stripping voltammetry was carried out between −0.30 to −0.60 V, using differential pulse voltammetry (DPV) with the following parameters: initial potential of −0.30 V; final potential of −0.60 V; amplitude of 0.05 V; potential incremental of 0.004 V; pulse width of 0.05 s; pulse period of 0.5 s; and quiet time of 2 s.

### Real sample collection and processing

The Dagujia River (121.27–121.35°E, 37.28–37.57°N) flows into the Huanghai Sea (China) and was chosen as a typical local coastal river. It is the second largest river in Yantai and known as the mother river of the Yantai region. All samples were collected during April (Spring) and September (Autumn), with the salient features of the samples assessed (Fig. 1, Table S1). All water samples were collected and stored in acid-cleaned polyethylene bottles, then hydrochloric acid was added to keep pH < 2, samples were filtered (0.45 μm membrane filters) and stored in the dark at 4 °C until determination.

### Determination of TFe and ADFe in coastal river water

**Figure 2:**
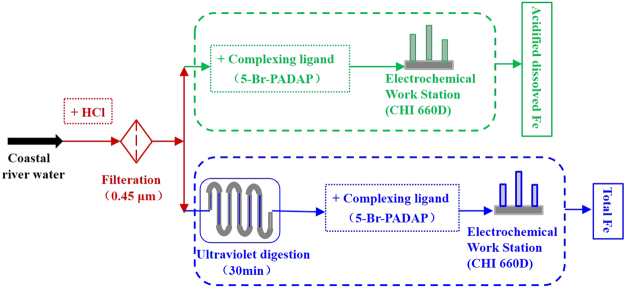
Process-diagram for the analysis of acidified dissolved Fe (ADFe) and total Fe (TFe). ADFe concentrations were obtained through electrochemical detection combined with complexing ligand after acidification and filtration processing. TFe concentrations were obtained through electrochemical detection combined with a complexing ligand after acidification, filtration and digestion processing.

TFe and ADFe were detected according to the summarized methodologies (Fig. 2), where water sample pre-treatments had three steps: acidification, filtration and digestion. Samples were acidified by the addition of 1 mL HCl per 100 mL water, adjusting the pH value to below 2.0. Acidification was utilized to leach the Fe in particulate matter and destroy complexes of Fe and organic matter, releasing unstable Fe complexes. For the filtration step, water samples were filtered using 0.45 μm cellulose acetate membranes to remove insoluble material. Samples pre-processed using acidification and filtering were analyzed by stripping voltammetry. Direct electrochemical analysis established the concentration of ADFe species, while TFe concentration was obtained in samples pre-processed with digestion as well as filtration and acidification^39^. ADFe includes Fe(II) and Fe(III) ion, organically and inorganically complexed Fe, colloidal Fe and particulate Fe, which are easily dissolved by acid. TFe is defined as the whole pool of Fe(II) and Fe(III) ion, organically and inorganically complexed Fe, colloidal Fe and particulate Fe. Voltammetric measurements were performed on water samples diluted to an appropriate concentration, with 0.1 M acetate buffer (pH 6.0) as the supporting electrolyte. All samples were measured three times in the experimental period and the mean of at least three determinations were used for subsequent analyses.

## Results and Discussion

### Method results and discussion

#### Choice of method

**Figure 3:**
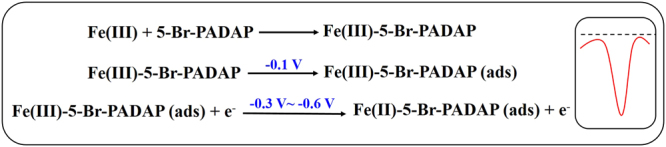
Schematic diagram for the detection of Fe(III)-5-Br-PADAP based on gold electrode.

A gold electrode combined with the 5-Br-PADAP was used for the analysis of Fe in coastal waters. Commercial gold electrodes exhibit good uniformity and reproducibility. In addition, the electrodes possess good sensitivity due to their unique electronic conductivity. More importantly, gold has been shown to have excellent electrochemical signals in response to Fe(III). 5-Br-PADAP was selected as the Fe-complexing agent as it contains −N = N and −OH groups as electron donor species^40^, forming chelate compounds with Fe at a ratio of 1:2 due to its high Fe binding affinity. 5-Br-PADAP was selected as it is very stable and can be used either in acidic or alkaline conditions, as well as the benefit of a short chelating reaction time (<3 min). During Fe determination, 5-Br-PADAP complexes with Fe(III) and the complexed compound is absorbed on the electrode surface with application of a negative potential. Then, during the voltammetric scan, Fe(III) is reduced to Fe(II), causing an apparent reductive peak in the current (Fig. 3).

Other electroanalytical methods using a complexing agent for the determination of Fe, are also shown in Table 1. A gold electrode modified with 2-Mercaptosuccinic acid (MSA) was reported for Fe determination^41^, showing a lower detection limit but also a narrow linear range and the self-assembly of MSA was time-consuming. Ligands that have been reported as successful for Fe determination, include PAN^32^, Triethanolamine (TEA)^42^, NN^33^, salicylaldoxime (SA) and DHN^34^. PAN- and TEA-based methods utilize bismuth film modified electrodes (BiF/GCE), which have the potential problem of bismuth instability. Additionally, PAN-based methods resulted in a pre-peak due to the presence of the ligand, affecting the limit of detection for Fe. The ligands NN and SA are commonly used in Competitive Ligand Exchange Cathodic Stripping Voltammetry (CLE-CSV) for the analysis of Fe speciation. NN-based methods suffer from an underlying peak in Fe concentrations below 0.5 nM. DHN-based methods exhibit superior sensitivity with mercury drop electrodes only, with a reported Hanging Mercury Drop Electrode (HMDE) and 5-Br-PADAP method achieving good Fe detection^43^. However, when considering the toxicity of mercury, the method presented in this study using an Au electrode and 5-Br-PADAP, is more practically applicable for the determination of Fe. As shown in Table 1, a relatively wide linear range, shorter reaction time and a more stable electrochemical performance could be obtained using this novel method. Considering the relatively high concentrations of Fe observed and the more complex environment in coastal river waters, this novel method may be valuable in the determination of Fe in coastal river samples.

**Table 1:** Comparison of different electroanalytical methods together with complex agents for determination of Fe(III) in the water samples.

Method	Electrode	Agents	Linear range (nM)	LOD (nM)	Reaction time (Min)	Reference
SWV	Au	MSA	0.1~ 6	0.03	~5	^42^
CSV	BiF/GCE	PAN	16~1070	1.78	Not mentioned	^32^
CSV	BiF/GCE	TEA	25~1000	7.7	Not mentioned	^43^
CLE-CSV	HMDE	NN	Not mentioned	1.80	Not mentioned	^33^
CSV	MDE	DHN	Not mentioned	1.20	>6	^34^
CSV	HMDE	5-Br-PADAP	Not mentioned	0.39	Not mentioned	^44^
CSV	Au	5-Br-PADAP	10~1000	1.20	<3	This work

SWV: Square Wave Stripping Voltammetry; CSV: Cathodic Stripping Voltammetry; CLE-CSV: Competitive Ligand Exchange Cathodic Stripping Voltammetry; BiF/GCE: bismuth Film Modified Electrode; HMDE: Hanging Mercury Drop Electrode; MED: Mercury Drop Electrode.

### Effects of accumulation potential and accumulation time

To optimize the stripping response, the accumulation potential and time of Fe(III)-5-Br-PADAP accumulation on the gold electrode, were investigated. Peak current increased from 0.223 μA to 0.348 μA when the potential varied from −0.3 V to −0.1 V (Fig. S1), which is probably due to the positively charged complex being strongly adsorbed on the gold electrode. The observed peak decreased gradually at a higher potential value, as insufficient Fe(III)-5-Br-PADAP complex was adsorbed at the gold electrode, therefore, the optimal accumulation potential was chosen to be −0.1 V. The effect of accumulation time on the peak current was studied ranging from 180 to 540 s (Fig. S2), with the complex peak current increasing rapidly from 0.212 μA to 0.303 μA as accumulation time increased to 360 s, then tending to increase slowly for the slope of the line ranging from 360 to 540 s becoming smaller. Considering the time taken for analysis and high level of energy consumption, the optimal accumulation time was selected to be 360 s.

### The calibration curve for determination of Fe

**Figure 4:**
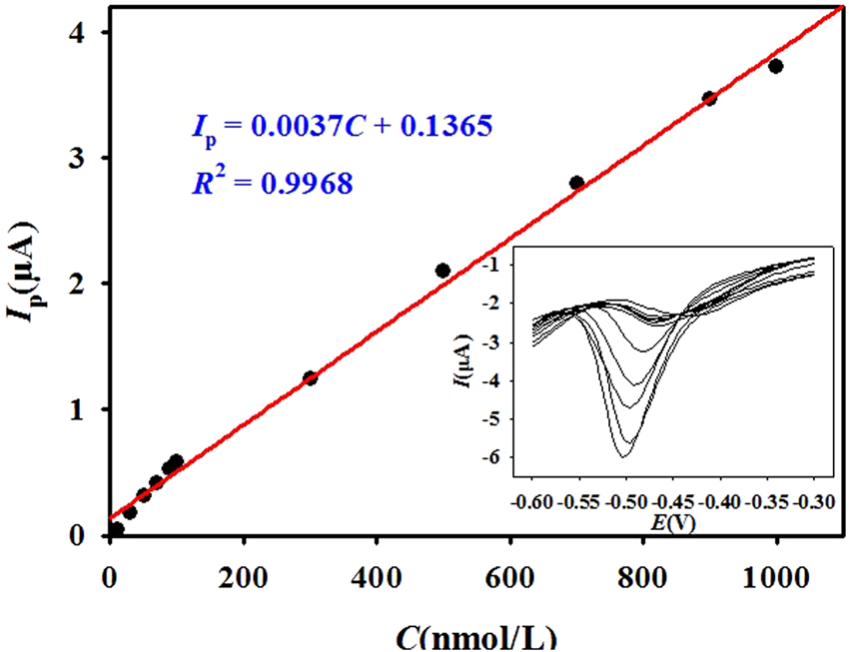
Calibration curve for Fe(III) at the gold electrode surface, where the concentrations of Fe(III) range from 0.01 μM to1 μM in 0.1 M acetate buffer (pH 6.0) containing 20 μM 5-Br-PADAP. Inset shows the voltammetric responses of Fe(III).

The calibration curve for the determination of Fe(III) at the gold electrode was established under optimal conditions of an accumulation potential of −0.1 V and an accumulation time of 360 s (Fig. 4). The linear calibration curve for Fe(III) was obtained from 0.01 μM to 1 μM and could be expressed by the regression equation: *I*_p_ = 0.0037*C* + 0.1365 (*R*^2^ = 0.9968). The sensitivity and detection limit established for Fe(III) determination were 3.7 nA nM^−1^ and 1.2 nM (S/N = 3), respectively. This method showed a relatively superior performance to the comparative method, such as higher sensitivity, relatively lower detection limit, wider linear range, more straight-forward analytical procedure and low-cost instrumentation and analytical procedure. These results indicate that this method may be a beneficial and suitable method for Fe determination.

### Method validation

**Figure 5:**
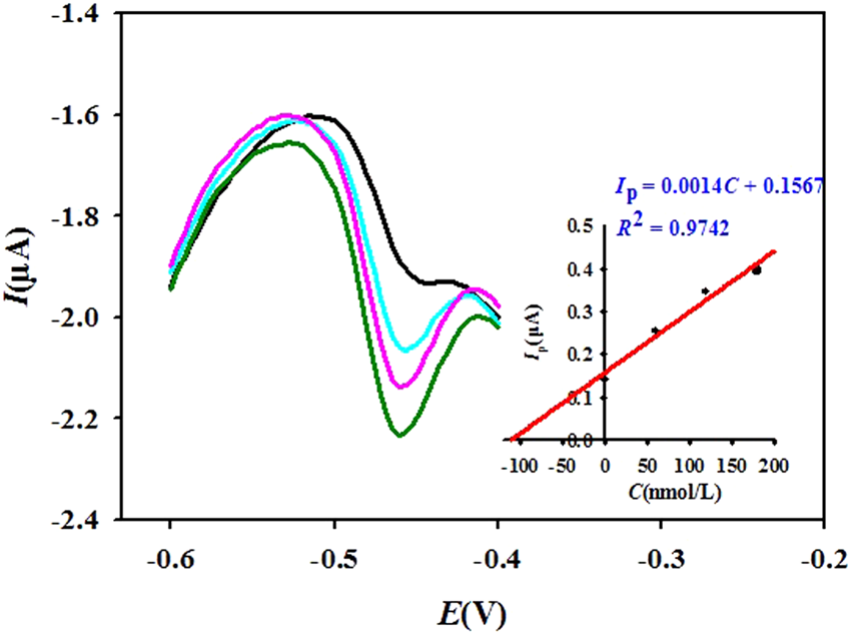
Voltammetric responses of Fe(III) in the water quality assessment of the Fe standard sample, by the standard addition method. The concentrations of added Fe(III) were 0, 60, 120 and 180 nM (from top to bottom), respectively. Inset shows the fitted curve for Fe(III) concentration determination.

Considering the accuracy and effectiveness of the proposed method, detection and quantification of the concentration had been done against standard reference material and standard method^44^. The Fe standard reference material was the GSB 07-1188-2000 standard, with the voltammetric responses of Fe(III) in the standard sample and coastal water samples tested by the standard addition method (Fig. 5). The inset in Fig. 5 shows the fitted curve for Fe(III) determination. The concentration of Fe(III) detected in all Dagujia River samples (diluted using 0.1 M acetate buffer) by our proposed method, was compared to the Fe-phenanthroline Spectrophotometry method which is the China National Standard Method for determination of Fe. At least three determinations were performed for all analyses and the certified values of iron standard sample and the real samples tested by spectrophotometry are shown in Table 2. These results indicate that this novel method is reliable and suitable for determination of Fe(III) in complex natural coastal waters.

**Table 2:** Comparison of this method and standard value for determination of Fe(III) in the water samples.

Water Samples	Detected by this method (nM)	Certified value (nM)
The Fe standard sample (GSB 07-1188-2000)	112 ± 3.8	115
Sample 1 of Dagujia River (April)	197 ± 4.3	198
Sample 2 of Dagujia River (April)	90 ± 3.5	87
Sample 3 of Dagujia River (April)	122 ± 4.2	124

The validation of this method was performed by comparing the concentration of TFe established by both methods, while comparison of the quantification of ADFe was not possible. The difference between TFe and ADFe samples was the pretreatment processes applied, with the sum of ADFe found to be proportional to TFe concentrations using the novel method, confirming its accuracy for ADFe analysis.

### Environmental results and discussion

**Figure 6:**
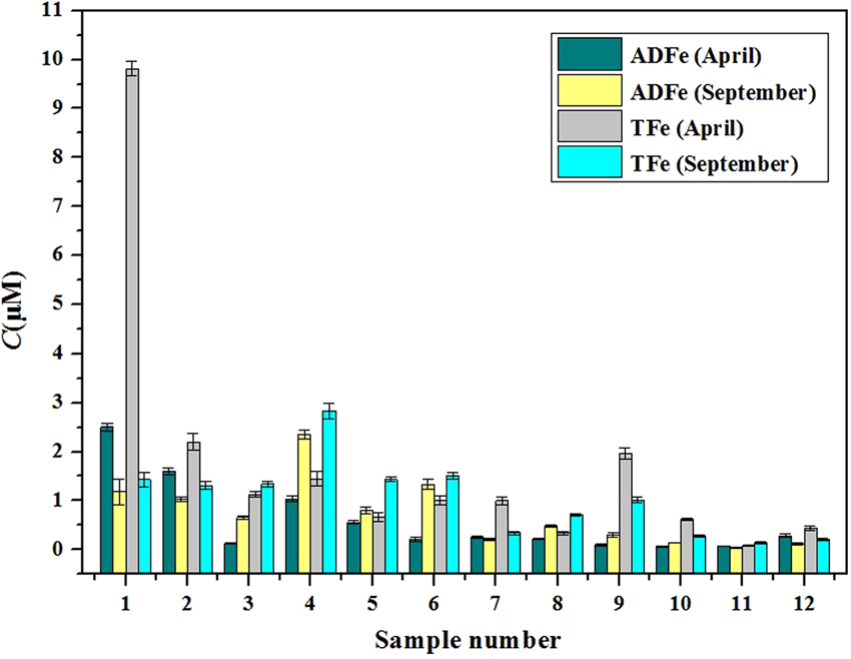
The concentrations of acidified dissolved Fe (ADFe) and total Fe (TFe), in samples from the Dagujia River (Yantai, China).

This novel method was applied to the determination of Fe concentrations in Dagujia River stations during April (spring) and September (autumn). Water sample characteristics and parameters (Table S1), as well as the concentration of various Fe species were obtained (Fig. 6). Additionally, the relationships between the concentration of Fe with DO and salinity were also studied and discussed.

Figure 6 shows the concentrations of ADFe and TFe in samples from the Dagujia River. In April, ADFe concentrations ranged from 0.06 to 2.51 μM, with an average concentration of 0.58 μM. The maximum ADFe concentration observed was at station 1 (2.51 μM), while the minimum concentration observed was at station 10 (0.06 μM). Meanwhile, TFe concentrations ranged from 0.09 to 9.82 μM, with average TFe concentrations of 1.73 μM. The maximum TFe concentration was observed at station 1 (9.82 μM), while the minimum concentration observed was at station 11 (0.09 μM). It was notable that the concentration of TFe at station 1 in the Dagujia River was relatively high, which may be caused by anthropogenic emissions. The relative concentrations of ADFe speciation which account for TFe concentrations were also investigated. Data shows that the abundance of ADFe may be significantly driven by the type and concentration of organic speciation and matter present. At station 1, 3, 6, 7 and 9, significant differences were observed in the concentrations of ADFe and TFe. The percentage of ADFe contributing to TFe was 25.0%, at its lowest reaching 5.3%. Conversely, at stations 2, 4, 5, 8, 11 and 12, the percentage contribution of ADFe were larger than 60.0% in TFe, with the largest percentage reaching 83.6%. ADFe accounted for a small part of the TFe, illustrating that the aquatic environment contained a large number of dissolved organic ligands. Evidence is accumulating for an association of Fe with both small, well-defined ligands, such as siderophores and with large, macromolecular complexes like humic substances, exopolymeric substances and transparent exopolymers^45^. When considering the type of dissolved organic material present in river environments, much of the dissolved organic material is likely to form stable complexes with Fe. Those complexes may be difficult to destroy by acid digestion, with the percentage of inert Fe therefore being higher. When little difference was observed between ADFe and TFe concentrations, it implies that acidification destroys most of the dissolved organic material and releases the contained Fe. The proportion of ADFe released is therefore, decided by the content and varieties of dissolved organic ligands in complex environmental water samples. In the September sampling event, ADFe concentrations ranged from 0.04 to 2.35 μM, with average ADFe concentration of 0.72 μM. The maximum concentration observed was at station 4 (2.35 μM), while the minimum concentration observed was at station 11 (0.04 μM). September TFe concentration ranged from 0.14 to 2.83 μM, with average TFe concentrations of 1.05 μM. The maximum concentration observed was at station 4 (2.83 μM), while the minimum concentration observed was at station 11 (0.14 μM). Concentrations of ADFe in April and September were almost the same, except for at stations 1, 3, 4 and 6. Seasonal changes affect the aquatic environment, resulting in variation in the type and concentration of organic matter present. For TFe, the concentration was almost constant between April and September, except for stations 1 and 4. The variation of concentration of TFe was likely caused by aspects such as surface runoff, atmospheric precipitation and human activity. The variation in concentrations of TFe and ADFe in the Dagujia River during April and September should be investigated further by seasonal monitoring. By analyzing the proportion of ADFe in TFe, water environment predictions might be improved, as well as our understanding of the biogeochemical Fe cycle.

**Figure 7:**
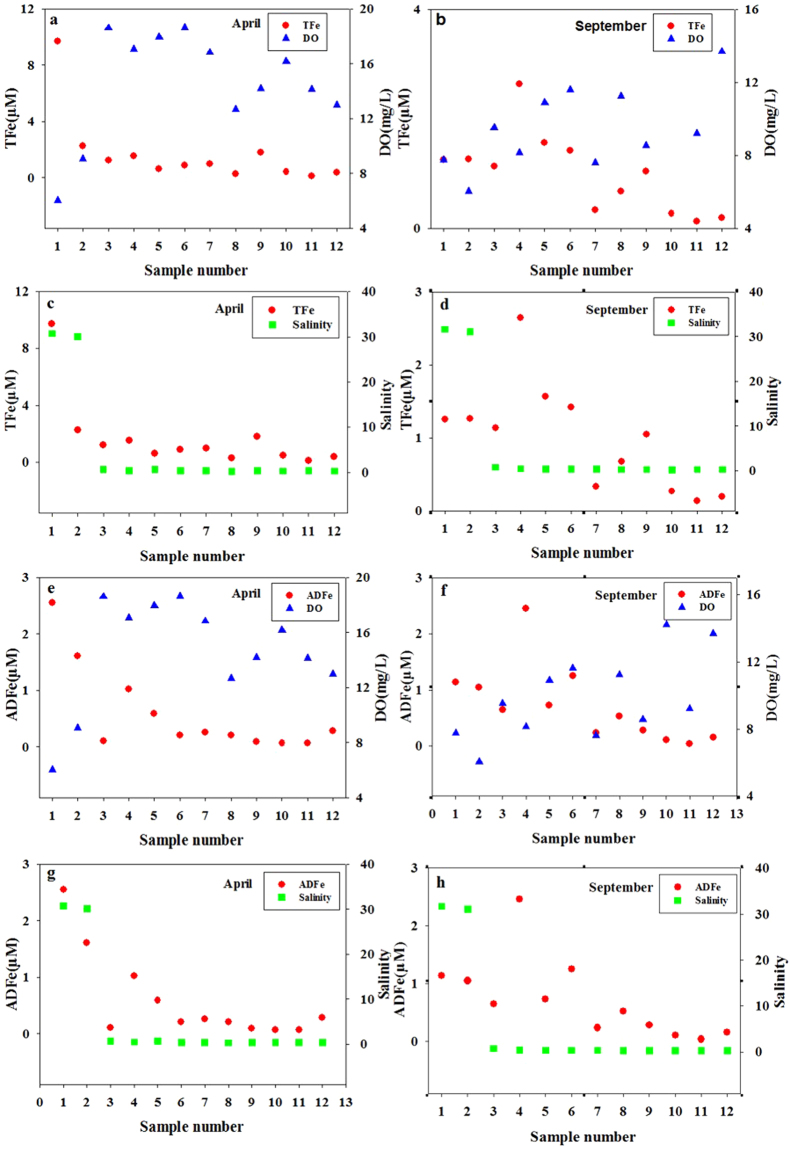
The relationship between the concentrations of the total Fe (TFe) with dissolved oxygen (DO) in April (**a**) and in September (**b**); the relationship between concentrations of TFe with salinity in April (**c**) and in September (**d**); the relationship between concentrations of acidified dissolved Fe (ADFe) with DO in April (**e**) and in September (**f**); the relationship between concentrations of ADFe with salinity in April (**g**) and in September (**h**).

The concentration of Fe in 12 sample stations during April (spring) and September (autumn) were established. Figure 7 shows the relationships between the concentrations of ADFe and TFe, with DO and salinity in April and September. The relationships between the concentrations of ADFe, TFe and DO were found to be negatively correlated (Fig. 7a,b,e,f), possibly as the content of DO was higher and Fe ions existed in high valence states, resulting in the formation of insoluble ferric hydroxide precipitates. Analyses and calculations show that in April, significant negative relationships were observed between DO and ADFe (r =−0.744, p < 0.01) and TFe (r =−0.704, p < 0.05). The correlation between the concentrations of ADFe, TFe and DO in September was relatively low, with r = 0.126, 0.167 respectively. No observed relationship between concentrations of ADFe or TFe and salinity (Fig. 7c,d,g,h). Coastal river areas are known to be greatly influenced by human activities, with complex environmental conditions^46^, resulting in the unclear relationships between Fe and many other parameters.

## Conclusion

In summary, an effective electrochemical method is proposed, for the sensitive determination of Fe, based on the use of a gold electrode and 5-Br-PADAP as the complexing agent. This method was calibrated by determining the certified water quality values for Fe standard sample and natural samples from the Dagujia River. Results indicate that this novel method is reliable and suitable for the determination of Fe(III) in waters from coastal zone areas. Furthermore, Fe species determination in coastal river water samples were successfully performed. This novel method is simple, reliable and exhibits high sensitivity and accuracy, providing an important method for the technical analysis of the distribution and speciation of Fe in coastal waters. Further research using this method will allow further understanding of the biogeochemical cycling of Fe in coastal zone.

## Electronic supplementary material

Supplementary Information

## References

[1] Morel FMM, Price NM (2003). The biogeochemical cycles of trace metals in the oceans. Science.

[2] Sunda WG, Huntsman SA (1995). Iron uptake and growth limitation in oceanic and coastal phytoplankton. Mar. Chem..

[3] Wedepohl KH (1995). The composition of the continental crust. Geochim. Cosmochim. Ac..

[4] Taylor SR (1964). Abundance of chemical elements in the continental crust: a new table. Geochim. Cosmochim. Ac..

[5] Laglera LM, Santos J, Caprara S, Monticelli D (2013). Quantification of iron in seawater at the low picomolar range based on optimization of Bromate/Ammonia/Dihydroxynaphtalene system by catalytic adsorptive cathodic stripping voltammetry. Anal. Chem..

[6] Zhu X (2017). Seasonal distribution of dissolved iron in the surface water of Sanggou Bay, a typical aquaculture area in China. Mar. Chem..

[7] Lu M, Rees NV, Kabakaev AS, Compton RG (2012). Determination of Iron: Electrochemical Methods. Electroanalysis.

[8] Guan J (2015). Flux characteristics of total dissolved iron and its species during extreme rainfall event in the midstream of the Heilongjiang River. J. Environ. Sci-China..

[9] Su H, Yang R, Zhang A, Li Y (2015). Dissolved iron distribution and organic complexation in the coastal waters of the East China Sea. Mar. Chem..

[10] Bergquist BA, Boyle EA (2006). Iron isotopes in the Amazon River system: Weathering and transport signatures. Earth Planet. Sc. Lett..

[11] Martin JH, Fitzwater SE (1988). Iron deficiency limits phytoplanktongrowth in the north-east Pacific subarctic. Nature.

[12] Levshina SI (2012). Iron distribution in surface waters in the Middle and Lower Amur basin. Water Resour..

[13] Liaghati T, Cox ME, Preda M (2005). Distribution of Fe in waters and bottom sediments of a small estuarine catchment, Pumicestone Region, southeast Queensland, Australia. Sci. Total Environ..

[14] Worsfold PJ, Lohan MC, Ussher S, Bowie AR (2014). Determination of dissolved iron in seawater: A historical review. Mar. Chem..

[15] Walworth NG (2016). Mechanisms of increased Trichodesmium fitness under iron and phosphorus co-limitation in the present and future ocean. Nat. Commun..

[16] Ruengsitagoon W (2008). Reverse flow injection spectrophotometric determination of iron(III) using chlortetracycline reagent. Talanta.

[17] Stookey LL (1970). Ferrozine–a new spectrophotometric reagent for iron. Anal. Chem..

[18] Grotti M, Abelmoschi ML, Soggia F, Frache R (2003). Determination of ultratrace elements in natural waters by solid-phase extraction and atomic spectrometry methods. Anal. Bioanal. Chem..

[19] Leao DJ, Junior MMS, Brandao GC, Ferreira SLC (2016). Simultaneous determination of cadmium, iron and tin in canned foods using high-resolution continuum source graphite furnace atomic absorption spectrometry. Talanta.

[20] Wu J, Boyle EA (1998). Determination of iron in seawater by high-resolution isotope dilution inductively coupled plasma mass spectrometry after Mg(OH)_2_ coprecipitation. Anal. Chim. Acta.

[21] Jong J, Schoemann V, Lannuzel D, Tison JL, Mattielli N (2008). High-accuracy determination of iron in seawater by isotope dilution multiple collector inductively coupled plasma mass spectrometry (ID-MC-ICP-MS) using nitrilotriacetic acid chelating resin for pre-concentration and matrix separation. Anal. Chim. Acta.

[22] Croot PL, Laan P (2002). Continuous shipboard determination of Fe(II) in polar waters using flow injection analysis with chemiluminescence detection. Anal. Chim. Acta.

[23] Bowie AR, Sedwick PN, Worsfold PJ (2004). Analytical intercomparison between flow injection-chemiluminescence and flow injection-spectrophotometry for the determination of picomolar concentrations of iron in seawater. Limnol. Oceanogr-Meth..

[24] Ussher SJ, Petrov I, Quéte CR, Worsfold PJ (2010). Validation of a portable flow injection–chemiluminescence (FI-CL) method for the determination of dissolved iron in Atlantic open ocean and shelf waters by comparison with isotope dilution-inductively coupled plasma mass spectrometry (ID-ICP-MS). Environ. Chem..

[25] Du Y (2013). Determination of iron(III) based on the fluorescence quenching of rhodamine B derivative. Talanta.

[26] Hu X, Pan D, Lin M, Han H, Li F (2015). Graphene oxide-assisted synthesis of bismuth nanosheets for catalytic stripping voltammetric determination of iron in coastal waters. Microchim. Acta.

[27] Li F (2015). Electrochemical determination of iron in coastal waters based on ionic liquid-reduced graphene oxide supported gold nanodendrites. Electrochim. Acta.

[28] Lin M, Pan D, Hu X, Li F, Han H (2015). A tin–bismuth alloy electrode for the cathodic stripping voltammetric determination of iron in coastal waters. Anal. Methods.

[29] Windmiller JR, Wang J (2013). Wearable Electrochemical Sensors and Biosensors: A Review. Electroanalysis.

[30] Caprara S, Laglera LM, Monticelli D (2015). Ultrasensitive and Fast Voltammetric Determination of Iron in Seawater by Atmospheric Oxygen Catalysis in 500 μL Samples. Anal. Chem..

[31] Huang Y, Yuan D, Zhu Y, Feng S (2015). Real-time redox speciation of iron in estuarine and coastal surface waters. Environ. Sci. Technol..

[32] Segura R, Toral MI, Arancibia V (2008). Determination of iron in water samples by adsorptive stripping voltammetry with a bismuth film electrode in the presence of 1-(2-piridylazo)-2-naphthol. Talanta.

[33] Nagai T, Imai A, Matsushige K, Yokoi K, Fukushima T (2004). Limnology.

[34] Van den Berg CMG (2006). Chemical speciation of iron in seawater by cathodic stripping voltammetry with Dihydroxynaphthalene. Anal. Chem..

[35] Merli D, Ravasio F, Protti S, Pesavento M, Profumo A (2014). ω-Thio nitrilotriacetic chemically modified gold electrode for iron determination in natural waters with different salinity. Talanta.

[36] Zhao J, Jin W (1989). A study on the adsorption voltammetry of the iron(III)-2-(5′-bromo-2′-pyridylazo) -5-diethylaminophenol system. J. Electroanal. Chem..

[37] Peng B (2015). Determination of total iron in water and foods by dispersive liquid-liquid microextraction coupled with microvolume UV-vis spectrophotometry. Food Chem..

[38] Zhu Y (2017). An electrochemical sensor based on reduced graphene oxide/gold nanoparticles modified electrode for determination of iron in coastal waters. Sensor. Actuat. B-Chem..

[39] Annibaldi A, Llluminati S, Truzzi C, Libani G, Scarponi G (2015). Pb, Cu and Cd distribution in five estuary systems of Marche, central Italy. Mar. Pollut. Bull..

[40] Rana S, Mittal SK, Kaur N, Banks CE (2017). Disposable screen printed electrode modified with imine receptor having a wedge bridge for selective detection of Fe(II) in aqueous medium. Sensor. Actuat. B-Chem..

[41] Reza KS, Abdolhamid HM, Asghar AF (2007). Sensitive determination of iron(III) by gold electrode modified with 2-mercaptosuccinic acid self-assembled monolayer. Anal. Chim. Acta.

[42] Bobrowski A, Nowak K, Zarebski J (2005). Application of a bismuth film electrode to the voltammetric determination of trace iron using a Fe(III)-TEA-BrO_3_^−^ catalytic system. Anal. Bioanal. Chem..

[43] Ghoneim MM, Hassanein AM, Hammam E (2000). Simultaneous determination of Cd, Pb, Cu, Sb, Bi, Se, Zn, Mn, Ni, Co and Fe in water samples by differential pulse stripping voltammetry at a hanging mercury drop electrode. Fresenius J. Anal Chem..

[44] Zakharova EA, Elesova EE, Noskova GN, Lu M, Compton RG (2012). Direct voltammetric determination of total iron with a gold microelectrode ensemble. Electroanalysis.

[45] Gledhill M, Buck KN (2012). The organic complexation of iron in the marine environment: a review. Front. Microbiol..

[46] Bose R, De A, Sen G, Mukherjee AD (2012). Comparative study of the physico-chemical parameters of the coastal waters in rivers Matla and Saptamukhi: impacts of coastal water coastal pollution. J. Water Chem. Techno..

