# Supplementary material for: Speciation determination of iron and its spatial and seasonal distribution in coastal river
Source: Sci Rep. 2018 Feb 7;8:2576. doi: 10.1038/s41598-018-20991-0 (PMC5803190; doi:10.1038/s41598-018-20991-0)
Supplement: Supplementary file 1 — Supplementary Information [file 41598_2018_20991_MOESM1_ESM.doc]

**Speciation determination of iron and its spatial and seasonal distribution in coastal river**

**Yun Zhu^1,2^, Xueping Hu^1,2^, Dawei Pan^1,2^, Haitao Han^1^, Mingyue Lin^1,2^, Yan Lu^1,2^, Chenchen Wang^1^ & Rilong Zhu^3^**

^1^ Key Laboratory of Coastal Environmental Processes and Ecological Remediation, Yantai Institute of Coastal Zone Research, Chinese Academy of Sciences, Yantai 264003, P.R. China

^2^ University of Chinese Academy of Sciences, Beijing 100049, P.R. China

^3^ Hunan Environmental Monitoring Center Station, State Environmental Protection Key Laboratory of Monitoring for Heavy Metal Pollutants, Changsha 410019, P.R. China

Yun Zhu and Xueping Hu contributed equally to this work.

Correspondence and requests for materials should be addressed to D. Pan (email: dwpan@yic.ac.cn) or R. Zhu (email: zrlden@hotmail.com)

Corresponding author：Dawei Pan

Tel.:+86 535 2109155; fax: +86 535 2109155.

E-mail addresses: [dwpan@yic.ac.cn](mailto:dwpan@yic.ac.cn) (D. Pan).

Corresponding author：Rilong Zhu

E-mail addresses: zrlden@hotmail.com (R. Zhu).

**Table S1. Location and salient features of the samples in Dagujia River of Yantai, China.**

| **Sample number** | **Location** | **T** **(°C)** | **DO (mg/L)** | **Salinity** | **C (us/cm)** |
| --- | --- | --- | --- | --- | --- |
| 1(April) | 121.30°E, 37.57°N | 15.8 | 6.04 | 30.75 | 38899 |
| 1(September) |  | 19.9 | 7.76 | 31.70 | 43559 |
| 2(April) | 121.29°E, 37.56°N | 16.5 | 9.07 | 30.12 | 38804 |
| 2(September) |  | 18.0 | 6.04 | 31.10 | 40799 |
| 3(April) | 121.29°E, 37.53°N | 18.1 | 18.61 | 0.64 | 1099 |
| 3(September) |  | 21.0 | 9.53 | 0.74 | 1280 |
| 4(April) | 121.28°E, 37.51°N | 21.7 | 17.08 | 0.44 | 841 |
| 4(September) |  | 18.4 | 8.15 | 0.42 | 739 |
| 5(April) | 121.30°E, 37.47°N | 21.9 | 17.97 | 0.72 | 1339 |
| 5(September) |  | 21.7 | 10.90 | 0.37 | 703 |
| 6(April) | 121.33°E, 37.43°N | 21.8 | 18.64 | 0.37 | 704 |
| 6(September) |  | 21.0 | 11.62 | 0.35 | 635 |
| 7(April) | 121.35°E, 37.42°N | 21.1 | 16.84 | 0.40 | 759 |
| 7(September) |  | 21.1 | 7.62 | 0.34 | 652 |
| 8(April) | 121.34°E, 37.39°N | 19.5 | 12.67 | 0.28 | 643 |
| 8(September) |  | 20.5 | 11.25 | 0.30 | 559 |
| 9(April) | 121.33°E, 37.36°N | 19.5 | 14.20 | 0.35 | 650 |
| 9(September) |  | 21.0 | 8.56 | 0.27 | 519 |
| 10(April) | 121.33°E, 37.33°N | 20.7 | 16.20 | 0.33 | 614 |
| 10(September) |  | 22.3 | 14.24 | 0.26 | 516 |
| 11(April) | 121.30°E, 37.31°N | 19.4 | 14.14 | 0.38 | 706 |
| 11(September) |  | 21.5 | 9.22 | 0.30 | 570 |
| 12(April) | 121.27°E, 37.28°N | 20.4 | 13.00 | 0.34 | 626 |
| 12(September) |  | 20.7 | 13.71 | 0.31 | 579 |

**Figure S1.** Effect of the accumulation potential of gold electrode on the reduction current response of Fe(III)-5-Br-PADAP. Conditions: a supporting electrolyte of 0.1 M acetate buffer (pH 6.0) containing 100 nM Fe(III), 20 μM 5-Br-PADAP.

**
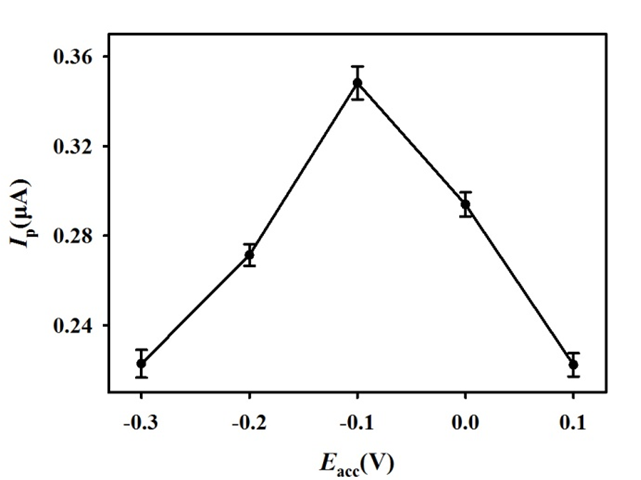
**

**Figure S2.** Effect of the accumulation time of gold electrode on the reduction current response of Fe(III)-5-Br-PADAP. Conditions: a supporting electrolyte of 0.1 M acetate buffer (pH 6.0) containing 100 nM Fe(III), 20 μM 5-Br-PADAP.

**
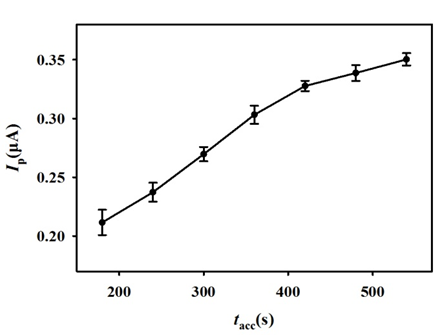
**
